# Quality of Life and Hormonal Impairment in Pediatric Patients With Craniopharyngiomas

**DOI:** 10.7759/cureus.52621

**Published:** 2024-01-20

**Authors:** Bárbara Pereira Neto, Ines Pais Cunha, Ana Laura Leite-Almeida, Sofia Ferreira, Janine Coelho, Rita Lago, Josué Pereira, Maria João Gil-da-Costa, Paulo Almeida, Cintia Castro-Correia

**Affiliations:** 1 Pediatrics, Centro Hospitalar Universitário de São João, Porto, PRT; 2 Pediatric Endocrinology and Diabetology Unit, Centro Hospitalar Universitário de São João, Porto, PRT; 3 Pediatric Oncology, Centro Hospitalar Universitário de São João, Porto, PRT; 4 Radiotherapy, Centro Hospitalar Universitário de São João, Porto, PRT; 5 Pediatric Neurosurgery, Centro Hospitalar Universitário de São João, Porto, PRT; 6 Neurosurgery, Faculdade de Medicina da Universidade do Porto, Porto, PRT; 7 Pediatric Neurosurgery-Neuroscience Center, CUF Hospital, Porto, PRT; 8 Psychology, Centro Hospitalar Universitário de São João, Porto, PRT; 9 Psychology, ISMAI (Instituto Universitário da Maia), Maia, PRT; 10 Pediatric Endocrinology and Diabetology Unit, Centro Hospitalar Universitário São João, Porto, PRT; 11 Gynecology-Obstetrics and Pediatrics, Faculdade de Medicina da Universidade do Porto, Porto, PRT

**Keywords:** oncology pediatrics, hypopituitarism, endocrine disorders, quality-of-life, craniopharyngioma

## Abstract

Introduction: Craniopharyngiomas (CP) are tumors in the sellar region that, despite a high survival rate, are associated with significant morbidity, including hypothalamic, hormonal, and visual dysfunction. This study aimed to assess the quality of life (QoL) in pediatric patients with CP and to evaluate its relationship with various factors, with a focus on the impact of endocrine dysfunction.

Methods: In this observational cross-sectional study, patients with CP aged between 0 and 18 years, currently followed up in a tertiary hospital by a multidisciplinary team, were included. QoL was assessed using the validated PEDS-QL4.0 questionnaire, which was administered to parents. This tool estimates Global QoL (QoL-G), further divided into Physical (QoL-P) and Psychosocial (QoL-PS) dimensions, including Emotional (QoL-Em), Social (QoL-S), and School (QoL-Sc) aspects. In Portugal, the estimated average QoL-G is 79.8, QoL-P is 83.5, and QoL-PS is 78.2. Variables studied included gender, current and diagnostic age, follow-up time, presence of hydrocephalus, hypothalamic involvement, type of resection (total or subtotal), radiotherapy, visual impairment, hormonal deficits, and therapy.

Results: The study included 11 patients with a median age of 15.2 years (interquartile ratio (IQR), 9.7-17.9 years) and a mean age at diagnosis of 9.3±4.1 years. Of these patients, 54.5% were male, and 36.4% were obese. Subtotal resection was performed in 72.7% of cases. Hydrocephalus was present in 54.5% of the patients, hypothalamic involvement in 63.7%, radiotherapy was received by 81.8%, and visual impairment was noted in 54.5%. All patients presented with at least one hormonal deficit. The average QoL-G was 69.9±22.5, with QoL-P at 66.9±30.0 and QoL-PS at 70.9±21.4. A worse QoL-S was associated with female gender (p=0.030) and subtotal resection (p=0.048). Worse QoL-G, QoL-P, QoL-Em, and QoL-PS were linked to hypothalamic involvement (p values 0.008, 0.025, 0.015, and 0.009, respectively). Irradiated patients had worse QoL-G (p=0.006). Treatment with sexual hormones enhanced QoL-Global (p=0.035) and QoL-Emotional (p=0.020), while treatment for adrenal insufficiency and diabetes insipidus improved QoL-Emotional (p=0.021 and p=0.013). No significant associations with visual deficit or obesity were found.

Conclusions: Pediatric patients with CP appear to have poorer QoL-G, QoL-P, and QoL-PS compared to the healthy Portuguese population. However, the small sample size limits statistically significant associations with many of these variables. Predictors of worse QoL include female gender, hypothalamic involvement, subtotal resection, and radiotherapy. The results may be biased due to the small sample size, questionnaire administration to parents, and possible inadequacy of the questionnaire for the studied population. There is a need for a more suitable tool to enable a more precise assessment of QoL in these patients.

## Introduction

Craniopharyngiomas (CP) are tumors of the sellar region associated with high survival rates but substantial morbidity [1-3]. Despite their low-grade histological malignancy [4], these tumors frequently impact critical areas like the pituitary, infundibulum, hypothalamus, and limbic system, significantly affecting patients' quality of life (QoL) [1]. This impact is not only due to the extent of the tumor itself but also to the iatrogenic effects resulting from surgical resection [3,5]. Long-term morbidity primarily stems from hypothalamic and visual dysfunction, hormonal deficits, and the consequences of radiotherapy and surgical approaches [3]. Previous reports assessing psychosocial and physical functioning have shown a range of outcomes from impaired to excellent [6-8], with psychosocial aspects appearing to be more adversely rated than the physical [4,6-10]. Factors previously associated with worse QoL include younger age at diagnosis, pre-operative functional impairment, tumor size, hypothalamic and third ventricle involvement at presentation, treatment with surgery alone, tumor recurrence, and endocrine, neurological, and ophthalmological sequelae [1,4,6,8,10,11].

This study aims to assess the QoL in pediatric patients diagnosed with CP and to examine its relationship with various factors, including hypothalamic involvement, visual acuity, obesity, type of surgery, radiotherapy, and especially hormonal deficits and hormone replacement therapy.

## Materials and methods

We conducted a cross-sectional observational study that included all patients up to 18 years old diagnosed with childhood-onset CP, followed at the Endocrinology ambulatory clinic of a Portuguese tertiary hospital. Hospital digital database information was analyzed, after receiving approval from the hospital’s Ethics Committee (Comissão de Ética do Centro Hospitalar Universitário de São João/Faculdade de Medicina da Universidade do Porto (number 132/2023)).

Variables considered for analysis encompassed age, gender, body mass index (BMI), age at diagnosis, hypothalamic involvement, hydrocephalus at diagnosis, type of surgery, time since surgery, radiation therapy, duration of follow-up, current disease status, current visual impairment, hormonal deficits (hypothyroidism, growth hormone deficiency, adrenal insufficiency, diabetes insipidus, and hypogonadism), and hormonal replacement therapy.

The Pediatric Quality of Life Inventory (PEDS-QOL 4.0) questionnaire was administered to all patients. It was answered by their respective parents through phone or in-person interviews, after providing informed consent. This questionnaire assessed a Global Quality of Life score (QoL-G), which was further divided into Physical (QoL-P) and Psychosocial (QoL-PS) dimensions. The latter included Emotional (QoL-Em), Social (QoL-S), and School (QoL-Sc) aspects.

Nominal variables were expressed as n (%), and categorical variables were described as mean ± standard deviation (SD) or median (IQR - Q1, Q3), depending on the normality assessed by the Shapiro-Wilk test. To compare QoL scores in our population with the Portuguese population mean [12], we used a one-sample t-test or a one-sample Wilcoxon signed-rank test, based on variable normality. Associations between categorical and continuous variables were evaluated using independent t-tests or Mann-Whitney tests, depending on variable normality. Correlations between continuous variables were assessed using Spearman’s correlation test. The level of statistical significance was set at p<0.05.

## Results

Eleven patients were included in this study, with a median age of 15.2 (9.7-17.9) years, of whom six (54.5%) were male. The average BMI was 25.3±6.7 kg/m^2^, and four (36.4%) patients were classified as obese. The mean age at diagnosis was 9.3±4.1 years, with follow-up time averaging 5.3±3.2 years, and a mean time since resection of 5.2±3.3 years. Hypothalamic involvement was present in seven (63.7%) patients, nine (81.8%) underwent radiation therapy, and six (54.5%) exhibited visual impairment. All patients had at least two hormonal deficiencies, and in three (27.3%) patients, all hormonal axes were impaired. Ten (90.9%) patients had hypothyroidism (all treated with levothyroxine), 10 (90.9%) had growth hormone deficiency (of which four (44.4%) were currently receiving hormonal supplementation, and two (22.2%) had received it in the past), seven (63.6%) had adrenal insufficiency (all treated with hydrocortisone), nine (81.8%) had diabetes insipidus (all treated with desmopressin), and six (54.5%) had hypogonadism (of which four (66.7%) were receiving hormonal supplementation). Table 1 shows a summary of these descriptive variables.

**Table 1:** Cohort characterization Categorical variables: results expressed as number of patients and percentage between brackets. Continuous variables with symmetric distribution: results expressed as mean ± standard deviation (SD). Continuous variables with asymmetric distribution: results expressed as median (interquartile range, IQR).

Feature	Cohort (n=11)
Gender: Male, n (%)	6 (54.5)
Age (years), median (IQR)	15.2 (9.7–17.9)
BMI (kg/m^2^), mean ± SD	25.3±6.7
Weight classification	
Normal, n (%)	5 (45.5)
Overweight, n (%)	2 (18.2)
Obese, n (%)	4 (36.4)
Age at diagnosis (years), mean ± SD	9.3±4.1
Time of follow-up (years), mean ± SD	5.3±3.2
Hydrocephalus at diagnosis, n (%)	6 (54.5)
Hypothalamic involvement	7 (63.7)
Compression, n (%)	2 (18.2)
Invasion, n (%)	5 (45.5)
Radiotherapy, n (%)	9 (81.8)
Visual impairment, n (%)	6 (54.5)
Surgery: Subtotal resection, n (%)	8 (72.7)
Time since surgery (years), mean ± SD	5.2±3.3
Impaired hormonal axes, median (IQR)	4.0 (3.0–5.0)
At least two axes, n (%)	11 (100)
All axes, n (%)	3 (27.3)
Hormonal impairment, n (%)	11 (100)
Hypothyroidism, n (%)	10 (90.9)
Treated with levothyroxine, n (%)	10 (100)
GH deficiency, n (%)	10 (90.9)
Currently treated with GH, n (%)	4 (44.4)
Previously treated with GH, n (%)	2 (22.2)
Adrenal insufficiency, n (%)	7 (63.6)
Treated with hydrocortisone, n (%)	7 (100)
Diabetes insipidus, n (%)	9 (81.8)
Treated with desmopressin, n (%)	9 (100)
Hypogonadism, n (%)	6 (54.5)
Sexual hormone supplementation, n (%)	4 (66.7)

The QoL questionnaire showed a mean QoL-G score of 69.9±22.5, QoL-P score of 66.9±30.0, and QoL-PS score of 70.9±21.4 points (QoL-Em: 60.9±31.6; QoL-S: 90.0 (60.0 - 100.0); QoL-Sc: 85.0 (55.0-90.0)). Lower scores of QoL-G, QoL-P, QoL-Em, and QoL-PS were seen in this cohort when compared with the Portuguese pediatric population mean [12], although no statistically significant difference was found, as presented in Table 2.

**Table 2:** Comparison between the quality of life (QoL) assessed in this cohort of patients with craniopharyngioma and a healthy Portuguese pediatric cohort Continuous variables with symmetric distribution: Results expressed as mean ± standard deviation (SD); compared using the one-sample t-test. Continuous variables with asymmetric distribution: Results expressed as median (IQR); compared using the one-sample Wilcoxon signed-rank test.

	QoL assessed in patients with CF	QoL assessed in a healthy Portuguese pediatric cohort	p value
QoL-G	69.9 ± 22.5	79.8 ± 12.1	0.175
QoL-P	66.9 ± 30.0	83.5 ± 14.8	0.096
QoL-Em	60.9 ± 31.6	73.3 ± 16.7	0.221
QoL-S	90.0 (60.0 – 100.0)	84.6 ± 15.1	0.789
QoL-Sc	85.0 (55.0 – 90.0)	78.2 ± 15.9	0.593
QoL-PS	70.9 ± 21.4	78.2 ± 12.9	0.282

Variables significantly associated with lower QoL-G score were hypothalamic involvement (p=0.008), radiotherapy (p=0.006), and the absence of hormonal supplementation in patients with hypogonadism (p=0.035). Hypothalamic involvement was also significantly associated with lower QoL-P (p=0.025) and QoL-PS (p=0.009) scores. Subtotal resection (STR) was associated with lower QoL-P (p=0.002) and QoL-S (p=0.048) scores. Factors associated with a lower QoL-Em score included hypothalamic involvement (p=0.015), the absence of adrenal insufficiency (p=0.021), and diabetes insipidus (p=0.013), and the lack of hormonal supplementation in patients with hypogonadism (p=0.020). Additionally, female patients had a significantly lower QoL-S score (p=0.030). No variable was found to be significantly associated with QoL-Sc scores. A comprehensive summary of these associations is presented in Table 3.

**Table 3:** Association between different variables and each quality of life (QoL) score Continuous variables with symmetric distribution: Results expressed as mean ± standard deviation (SD); compared using the independent samples t-test. Continuous variables with asymmetric distribution: Results expressed as median (IQR); compared using the Mann-Whitney U test.

QoL Score	Variable	Yes		No		p-value
		n	Score	n	Score	
QoL-G	Male gender	6	80.9 ± 19.3	5	56.8 ± 20.1	0.073
	Obesity	4	65.2 ± 20.5	7	72.6 ± 24.7	0.626
	Hydrocephalus	6	80.2 ± 18.9	5	57.5 ± 21.5	0.095
	Hypothalamic involvement	7	57.7 ± 18.9	4	91.2 ± 4.6	0.008
	Subtotal resection	8	62.1 ± 21.7	3	90.6 ± 2.7	0.056
	Radiotherapy	9	65.0 ± 21.9	2	92.1 ± 0.6	0.006
	Visual impairment	6	65.4 ± 19.0	5	74.3 ± 27.3	0.493
	Adrenal insufficiency	7	76.1 ± 20.1	4	59.0 ± 25.0	0.243
	Diabetes insipidus	9	73.2 ± 23.5	2	54.9 ± 9.1	0.320
	GH supplementation	6	80.5 ± 18.8	4	63.1 ± 19.4	0.194
	Hypogonadism	6	76.0 ± 19.5	2	62.7 ± 41.0	0.530
	Sexual hormone supplementation	4	86.6 ± 12.5	2	54.9 ± 9.1	0.035
QoL-P	Male gender	6	81.7 ± 29.6	5	49.0 ± 20.7	0.067
	Obesity	4	57.0 ± 33.0	7	72.5 ± 29.2	0.439
	Hydrocephalus	6	77.6 ± 28.8	5	54.0 ± 29.0	0.211
	Hypothalamic involvement	7	52.4 ± 27.2	4	92.2 ± 13.7	0.025
	Subtotal resection	8	54.8 ± 26.1	3	99.0 ± 1.8	0.002
	Radiotherapy	9	59.8 ± 28.7	2	98.4 ± 2.2	0.101
	Visual impairment	6	61.4 ± 26.5	5	73.4 ± 35.8	0.540
	Adrenal insufficiency	7	71.0 ± 30.5	4	59.7 ± 32.3	0.578
	Diabetes insipidus	9	69.1 ± 32.6	2	56.9 ± 32.6	0.630
	GH supplementation	6	79.4 ± 30.9	4	58.6 ± 20.5	0.274
	Hypogonadism	6	79.9 ± 23.5	2	60.9 ± 50.8	0.691
	Sexual hormone supplementation	4	91.4 ± 17.2	2	56.9 ± 16.8	0.080
QoL-Em	Male gender	6	76.7 ± 16.6	5	42.0 ± 36.5	0.103
	Obesity	4	68.8 ± 14.9	7	56.4 ± 38.6	0.473
	Hydrocephalus	6	79.2 ± 18.3	5	39.0 ± 31.3	0.026
	Hypothalamic involvement	7	46.4 ± 30.0	4	86.3 ± 13.8	0.015
	Subtotal resection	8	54.4 ± 35.1	3	78.3 ± 7.6	0.105
	Radiotherapy	9	56.1 ± 33.2	2	82.5 ± 3.5	0.310
	Visual impairment	6	62.5 ± 25.4	5	59.0 ± 41.0	0.866
	Adrenal insufficiency	7	76.4 ± 17.3	4	33.8 ± 34.5	0.021
	Diabetes insipidus	9	71.1 ± 24.5	2	15.0 ± 7.1	0.013
	GH supplementation	6	68.3 ± 31.1	4	60.0 ± 32.4	0.693
	Hypogonadism	6	55.8 ± 35.8	2	50.0 ± 42.4	0.853
	Sexual hormone supplementation	4	76.3 ± 21.4	2	15.0 ± 7.1	0.020
QoL-S	Male gender	6	97.5(88.8-100.0)	5	60.0 (47.5-90.0)	0.030
	Obesity	4	87.5 (62.5-97.5)	7	90.0 (60.0-100.0)	0.700
	Hydrocephalus	6	92.5 (88.8-100.0)	5	60.0 (47.5-95.0)	0.177
	Hypothalamic involvement	7	85.0 (55.0-90.0)	4	97.5 (91.3-100.0)	0.054
	Subtotal resection	8	87.5 (56.3-90.0)	3	100.0	0.048
	Radiotherapy	9	90.0 (57.5-92.5)	2	100.0	0.055
	Visual impairment	6	87.5 (51.3-91.3)	5	100.0 (75.0-100.0)	0.094
	Adrenal insufficiency	7	90.0 (85.0-100.0)	4	75.0 (45.0-97.5)	0.441
	Diabetes insipidus	9	90.0 (72.5-100.0)	2	65.0	0.230
	GH supplementation	6	97.5 (81.3-100.0)	4	87.5 (51.3-90.0)	0.114
	Hypogonadism	6	92.5 (77.5-100.0)	2	80.0	1.000
	Sexual hormone supplementation	4	97.5 (91.3-100.0)	2	65.0	0.095
QoL-Sc	Male gender	6	85.0 (52.5-90.0)	5	65.0 (42.5-97.5)	1.00
	Obesity	4	62.5 (13.8-81.3)	7	90.0 (65.0-95.0)	0.107
	Hydrocephalus	6	85.0 (52.5-91.3)	5	65.0 (42.5-95.0)	0.792
	Hypothalamic involvement	7	65.0 (30.0-85.0)	4	90.0 (86.3-93.8)	0.071
	Subtotal resection	8	67.5 (36.3-93.8)	3	85.0	0.497
	Radiotherapy	9	70.0 (42.5-92.5)	2	87.5	0.477
	Visual impairment	6	67.5 (41.3-87.5)	5	90.0 (57.5-95.0)	0.464
	Adrenal insufficiency	7	85.0 (55.0-90.0)	4	75.0 (38.8-96.3)	0.155
	Diabetes insipidus	9	85.0 (42.5-90.0)	2	82.5	0.238
	GH supplementation	6	87.5 (77.5-92.5)	4	67.5 (16.3-88.8)	0.352
	Hypogonadism	6	85.0 (68.8-92.5)	2	60.0	0.615
	Sexual hormone supplementation	4	85.0 (73.8-88.8)	2	82.5	0.064
QoL-PS	Male gender	6	80.6 ± 16.1	5	59.3 ± 22.6	0.102
	Obesity	4	67.9 ± 16.3	7	72.6 ± 16.3	0.745
	Hydrocephalus	6	81.1 ± 16.6	5	58.7 ± 21.3	0.080
	Hypothalamic involvement	7	59.5 ± 18.1	4	90.8 ± 5.7	0.009
	Subtotal resection	8	64.6 ± 21.9	3	87.8 ± 3.8	0.021
	Radiotherapy	9	66.7 ± 21.4	2	90.0 ± 0.0	0.174
	Visual impairment	6	66.7 ± 19.4	5	76.0 ± 24.8	0.500
	Adrenal insufficiency	7	77.9 ± 17.4	4	58.8 ± 24.6	0.164
	Diabetes insipidus	9	74.6 ± 21.2	2	54.2 ± 17.7	0.239
	GH supplementation	6	80.8 ± 15.0	4	64.6 ± 22.4	0.203
	Hypogonadism	6	74.7 ± 19.8	2	63.3 ± 37.7	0.579
	Sexual hormone supplementation	4	85.0 ± 11.4	2	54.2 ± 17.7	0.055

Additionally, the number of affected hormonal axes exhibited a statistically significant and moderately positive correlation with QoL-G (p=0.047, r=0.608), and QoL-PS scores (p=0.029, r=0.653), as outlined in Table 4.

**Table 4:** Correlation between quality of life (QoL) score and the number of impaired hormonal axes Correlation between variables was assessed using Spearman's test.

QoL assessed with PEDS-QL	p-value	r
QL-G	0.047	0.608
QL-P	0.079	-
QL-Em	0.069	-
QL-S	0.056	-
QL-Ed	0.568	-
QL-PS	0.029	0.653

## Discussion

CP is a rare intracranial tumor originating from residual epithelial cells of Rathke’s pouch, typically located in the sellar/parasellar region, involving the third ventricle, optic chiasm, pituitary stalk, and hypothalamus [2]. Most cases have a suprasellar component [1], thereby increasing the risk of hormonal impairment [3].

They predominantly occur in pediatric patients but can also be diagnosed in adults and can be histologically categorized into adamantinomatous and papillary subtypes, the latter being infrequent in childhood-onset CP [1,4].

Despite being classified as low-grade histological malignancies [4], their proximity to crucial cerebral structures along the pituitary-hypothalamic axes makes them challenging to treat [1] without significantly compromising patients’ QoL [3]. Metastases and distant recurrences are rare and mainly iatrogenic [1].

CP diagnosis is often delayed due to nonspecific symptoms, initially manifesting as headache and nausea from hydrocephalus, followed by visual impairment [11] and endocrine deficiencies (present in up to 90% of patients [3]), leading to reduced growth rates and weight gain [1].

A multidisciplinary approach is essential to determine the optimal treatment course that balances survival and QoL [3,13]. Hypothalamic dysfunction, resulting from both the disease and its treatment, is a major risk factor for worse prognosis and QoL [1,3,6,14,15]. However, the impact of hypothalamic impairment on QoL seems to be more pronounced in the long-term follow-up [14].

Treatment options include surgery alone (gross total resection, GTR) or combining STR with adjuvant radiation [5,13]. Hypothalamic injury is a significant complication, with suprachiasmatic and hypothalamic involvement often associated with higher morbidity [3,5]. GTR remains the preferred approach when feasible [1,4], as STR has been linked to higher local recurrence rates in some studies [2]. However, depending on tumor location, STR may be the best option to optimize patients’ QoL by minimizing hypothalamic damage [2,4,9,13]. Two irradiation techniques may be used: photon-based radiotherapy and proton beam therapy. While there is limited data on the latter, it is believed to present less risk of neurological and neuroendocrine deficits [2] and secondary malignancies due to its more localized action [4]. Despite impacting visual and cognitive function, radiotherapy seems to have lower long-term toxicity than aggressive surgery [2], and the incidence of endocrine dysfunction has been lower than after GTR [3]. Thus, the current preferred treatment option to minimize related morbidity, albeit controversial, is STR followed by radiotherapy [1,4,5].

Tumor and iatrogenic sequelae encompass [1-3,13] hypothalamic syndrome, with obesity (the primary comorbidity, occurring despite adequate endocrine control and potentially exacerbated by other comorbidities limiting physical activity), decreased physical activity, sleep cycle dysregulation, autonomic dysfunction, and impaired appetite and satiety regulation. Hormonal deficits, whose rates usually increase after CP treatment; visual defects (usually present at diagnosis with potential to improve after surgery, but can also appear during treatment) and neurological symptoms (often temporary [4,6,16]) are also part of these sequelae. Neuroendocrine impairment, caused by both tumor extension and/or treatment consequences, affects QoL, primarily in social and emotional functioning [4,6,7,9], but also cognitive impairment, including executive function, attention, and episodic and working memory [1,4,6,13,15].

Previous studies have assessed QoL in CP patients, demonstrating a range of outcomes from impaired to excellent [6,7]. Identified risk factors for worse QoL include younger age at diagnosis, preoperative functional limitations, larger tumor volume, hypothalamic or third ventricle involvement, treatment with surgery alone, tumor recurrence, and endocrine, neurological, and ophthalmological sequelae [1,4,6,8,10,11].

In our cohort, we did not find statistically significant differences between the various QoL scores when compared to the average of the pediatric Portuguese population [12]. However, it is important to note that these scores tended to be lower in CP patients, and the statistical significance may be biased by the small sample size.

Specifically, we found that female patients with CP have significantly worse QoL-S scores than males, aligning with other studies that identified female sex as a predictor for depression [17] or poor QoL [3] in CP patients.

As expected, hypothalamic involvement was associated with lower QoL-G, QoL-P, and QoL-PS, particularly in the emotional aspect (QoL-Em).

Surprisingly, contrary to findings in other studies [2,3,14], in our cohort, both visual impairment and obesity were not significantly associated with lower QoL scores, although there was a tendency for these scores to be poorer.

STR was associated with lower QoL-P, QoL-S, and QoL-PS, while radiotherapy was related to lower QoL-G. It is essential to acknowledge that the combination of radiotherapy with STR has transformed CP management, treating residual tumors and avoiding radical surgeries associated with high morbidity [5]. Our finding is explained by the sample size, as the two patients not subjected to radiotherapy had excellent QoL scores, whereas those with poorer scores underwent radiotherapy. Among them, one is currently experiencing recurrence, another had a large tumor volume, and the last one had previously been diagnosed with severe intellectual impairment.

The presence of hydrocephalus was associated with better QoL-Em scores. This association can be attributed to two out of five patients with hydrocephalus also having significant intellectual disabilities. Additionally, one patient is currently experiencing recurrence.

The fact that most of our patients were under hormonal replacement limited the investigation of how endocrine dysfunction might impact QoL. Adrenal insufficiency and diabetes insipidus were associated with better QoL-Em scores, but it is important to note that all these patients are medicated, and this result may have been influenced by the comorbidities of this specific patient group. Supplementing sexual hormones in those patients with hypogonadism was linked to improved QoL-G and QoL-Em. Addressing delayed puberty, which has previously been associated with psychosocial dysfunction [3], can explain why children with this condition, when treated, may show better QoL scores, especially in the emotional aspect.

In our cohort, QoL-Sc did not show a significant association with any investigated variable, despite such associations being reported in previous studies [6]. Additionally, the number of affected hormonal axes demonstrated a statistically significant and moderately positive correlation with QoL-G and QoL-PS scores.

Despite these valuable insights, our study has some important limitations, namely, a small sample size due to the rarity of this disease, which limits the reliability of the observed associations. Additionally, we opted to administer questionnaires to parents to standardize responses across various age groups of patients. However, this may not fully capture the children's own perspectives on their QoL. Notably, previous studies have found that parental assessments of QoL in CP patients were lower than children’s own ratings [7,10]. Moreover, considering the unique characteristics and comorbidities of our study cohort, there is a possibility that the chosen questionnaire may not be the most suitable. Despite the existence of other questionnaires tailored to populations with hypopituitarism, all available options were designed for adults, leading us to choose PEDS-QOL 4.0. Further research should focus on providing a tool to assess the QoL of these pediatric patients with CP, considering their specific symptoms, complications, and limitations.

## Conclusions

Our findings suggest that children and young individuals with CP tend to have a lower quality of life than that of the healthy Portuguese population. However, the limited number of participants in our study hinders the establishment of statistically significant associations among many analyzed variables. We identified female gender, hypothalamic involvement, subtotal resection, and radiotherapy as potential predictors of a poorer quality of life. Nevertheless, these results might be influenced by the small sample size, the reliance on parental responses, and potential inadequacies in the applied questionnaire, which may not comprehensively capture the QoL specific to CP patients. Consequently, further research should aim to develop a more longitudinal and precise tool for assessing the QoL in this particular population.
